# 11q deletion in neuroblastoma: a review of biological and clinical implications

**DOI:** 10.1186/s12943-017-0686-8

**Published:** 2017-06-29

**Authors:** Vid Mlakar, Simona Jurkovic Mlakar, Gonzalo Lopez, John M. Maris, Marc Ansari, Fabienne Gumy-Pause

**Affiliations:** 1 CANSEARCH Research Laboratory, Geneva University Medical School, Avenue de la Roseraie 64, 1205 Geneva, Switzerland; 2 Department of Pediatrics, Onco-Hematology Unit, Geneva University Hospitals, Rue Willy-Donzé 6, 1205 Geneva, Switzerland; 3 Division of Oncology and Center for Childhood Cancer Research, Children’s Hospital of Philadelphia, Philadelphia, PA USA; 4 Department of Pediatrics, Perelman School of Medicine at the University of Pennsylvania, Philadelphia, PA USA

**Keywords:** Neuroblastoma, 11q deletion, MYCN amplification, Cancer, Development, Metastasis, Review

## Abstract

Deletion of the long arm of chromosome 11 (11q deletion) is one of the most frequent events that occur during the development of aggressive neuroblastoma. Clinically, 11q deletion is associated with higher disease stage and decreased survival probability. During the last 25 years, extensive efforts have been invested to identify the precise frequency of 11q aberrations in neuroblastoma, the recurrently involved genes, and to understand the molecular mechanisms of 11q deletion, but definitive answers are still unclear. In this review, it is our intent to compile and review the evidence acquired to date on 11q deletion in neuroblastoma.

## Background

Neuroblastoma (NB) is the most frequent extracranial solid tumour in children, accounting for 7 to 8% of all childhood malignancies and 15% of all cancer-related deaths in this population. It is the most frequently diagnosed cancer during infancy, the median age at diagnosis being about 19 months. While 90% of the patients are younger than 5 years, NB is very rare after the age of 10. NB originates from embryonal sympathoadrenal lineage of the neural crest and can arise anywhere along the sympathetic nervous system chain, with the majority of tumours occurring in the abdomen (65%), more particularly in the adrenal gland. The other common sites involved are chest, neck and pelvis. Regional lymph nodes, bone marrow, bone, liver and subcutaneous tissue are the most frequently observed metastatic localizations [1, 2].

NB is a complex and heterogeneous disease, with a marked variability in prognosis depending of the age, stage and biological characteristics at diagnosis. The clinical course ranges from spontaneous regression to inexorable progression and death despite multimodal treatments. NB differentiation into benign ganglioneuroma is not uncommon and known for a long time. While NB maturation occurs spontaneously or after chemotherapeutic treatments, multiple chemical modifiers were shown to suppress tumorigenicity and to induce NB differentiation in cell lines [3, 4].

Metastatic disease is present in approximately 50% of cases. Other biological factors associated with poor prognosis are: age at onset of more than 18 month at diagnosis, unfavourable histology, diploid DNA contents, *MYCN* amplification (MNA), and specific segmental chromosomal aberrations (SCA) such as 11q deletion, 1p deletion and 17q gain [1, 5, 6]. In contrast, an excellent overall (OS) and event-free survival in NBs with only numerical chromosome alterations is reported [7, 8]. In 2009, the International Neuroblastoma Risk Group Task Force published a classification system in order to stratify patients in different subgroups regarding the risk of death [9]. Recently, Matthay et al. proposed a modified version of this system taking into account the most recent genomic data and treatment approaches. Using clinical staging, image-defined risk factors for surgery, age at diagnosis, histology, tumour differentiation, *MYCN* status, genomic profile and ploidy, it is possible to stratify patients into four subgroups regarding the risk of death (very low, low, intermediate and high-risk). Whereas children in the very low-risk subgroup have an expected OS rate of 99–100%, patients in the high-risk subgroup have a rate of long-term survival of less than 50% despite dose-intensive, multimodal therapy including surgery, high dose chemotherapy with autologous bone marrow transplantation, radiotherapy and immunotherapy [10]. In regard to this poor outcome, it is obvious that new therapeutic strategies are required to improve the outcome and decrease the toxicity (for a more general review of Neuroblastoma authors recommend the recent review of Matthay et al.) [10].

For more than two decades, 11q deletion is known to be a recurrent genetic alteration and suspected to contain NB suppressor gene(s) [11–13]. While 11q deletion is more frequently detected than MNA (35–45% vs. 20–25%, respectively), both alterations are almost mutually exclusive [1, 5, 14–17]. Interestingly, 70 to 80% of stage 4 NBs have MNA or 11q deletion [18–20] and the poor prognosis significance of 11q deletion approaches that of MNA [5]. In this article, we will review the data concerning 11q deletion in order to have a deeper understanding of its role in the development and/or progression of NB. In addition, the signature of 11q-deleted genes will be analyzed using QIAGEN’s Ingenuity Pathway Analysis software (IPA, QIAGEN Redwood City) in order to understand potentially affected cellular processes and to position them in frequently dis-regulated networks of *MYCN*, MAPK and *TP53* pathways.

### Clinical implication of 11q aberrations in neuroblastoma

11q aberration is reported in 20 to 45% of NBs depending on the genetic alterations analysed and the screening method used [5, 15–17, 21–23]. While 11q23 is the most frequent 11q region found to be deleted, different studies screened allelic status of chromosome 11q using microsatellite markers [15, 21] and found 11q loss of heterozygosity (LOH) in approximately 34 to 44% of the NB samples. Nevertheless, a subgroup of these patients presented an unbalanced 11qLOH (unb11qLOH) defined as LOH at markers on 11q with retention of 11p material. Attieh et al. reported unb11qLOH in 17% of the whole NB group representing 50% of the LOH 11q23 NB, a proportion that increased with clinical risk. In this study, 11q23 LOH and unb11qLOH were both associated with high-risk features, but only unb11qLOH was independently associated with a decreased 3-year EFS (50% vs. 74%) [21]. These results confirmed previous published data, which have all suggested an association between LOH at 11q23, and high-risk NB features at stage 4 and unfavourable histology [11, 15, 17, 24–26].

Similar results were found by using fluorescence in situ hybridization (FISH). Spitz et al. reported 11q23 alterations in 26% of the 611 NB samples analysed with 18% displaying a deletion resulting in monosomy of distal 11q and 8% showing an imbalance with at least two intact copies of 11q23 with additional centromere copies. Interestingly, it was shown that the proportion of 11q alterations increased with stage (8% in stage 1, 10% in stage 2, 11% in stage 4S, 21% in stage 3 and 52% in stage 4) but also with age at diagnosis, proportion of 11q alteration increasing to more than 50% after the age of 2.5 years. As in other studies, 11q status was found prognostic for EFS and strongly correlated with occurrence of metastatic relapse [21, 27].

More recently, whole genome screening methods such as single nucleotide polymorphism (SNP) arrays and array based comparative genomic hybridization (CGH) have been used to detect large genomic gains or losses. High-resolution arrays were also able to detect recurrent small, interstitial, genomic alterations, possibly involved in the development of NB. While whole (or numerical) chromosome changes without segmental alterations (NCA) [8] are frequently observed in low risk NBs with good outcome, SCA defined by gains or losses of partial chromosome material are associated with poor prognosis in most cases [7, 8, 28, 29]. With losses of 1p, 3p, 4p and gains of 1q, 2p and 17q; 11q loss is one of the most frequent SCA observed, reported in 13 to 68% of the samples depending of the cohort analysed [5, 7, 8, 28, 30, 31]. As previously reported, 11q loss was more frequently found in NBs with high-risk (HR) features (47 to 68% of the HR vs. 47 to 50% for MNA) [30, 31], and regularly found associated with poor prognosis [5, 7, 8, 29, 32]. Schleiermacher et al. reported that in 147 NBs without MNA, a SCA profile was the strongest independent prognosis factor. In this cohort, 76% of NBs with SCAs showed an 11q deletion [8].

Interestingly, in the cohort studied by Caren et al., the median age at diagnosis was significantly higher in the 11q deletion group compared to NCA, MNA and 17q gain groups (42 months vs 3, 21 and 21 months, respectively). While the prognosis were similarly poor in MNA and 11q deletion groups (8 years OS ~35%), the median survival time after diagnosis was longer in 11q deleted NBs compared to MNA NBs (40 vs 16 months) [5]. Similar results concerning the age were recently reported in the Swedish cohort of unfavourable NBs where the median age at diagnosis was 58.5 months in NBs with 11q deletion vs 18 months in the MNA group [30]. While 11q alteration is detected mostly in older patients, over 18 months of age at diagnosis [31], recent analysis of the INRG database showed that in the youngest patients (< 18 months) with stage 3 NB, 11q deletion is the only factor found independently associated with poor EFS and OS [32].

Different studies, which analysed correlations between 11q deletion and other genetic abnormalities, reported not only an anti-correlation with MNA, but also a positive association with 17q gain and loss of 3p [5, 8, 16, 17, 31, 33]. More recently, 11q loss was found positively correlated with 4p loss and 7q gain but not with 17q gain [14]. Usually recognized as mutually exclusive, MNA and 11q deletion tumours were rarely reported [5, 15, 22, 27, 31, 34–36]. In these infrequent cases, the prognosis seems to be highly dramatic [27, 34, 35]. The clinical significance of intra-tumour heterogeneity of MNA (hetMNA) defined by the coexistence of MNA cells as well as non-MNA cells detected by FISH in the same tumour, was recently studied by Bogen et al. [34]. In older patient (> 18 months), tumours were mostly found di- or tetra-ploid containing lower number of MNA cells but higher number of SCA, including 11q deletion (4/10), and chromosomal breakpoints. Clinically, these patients often present an aggressive disease with tumour progression and relapse [34].

**Fig. 1:**
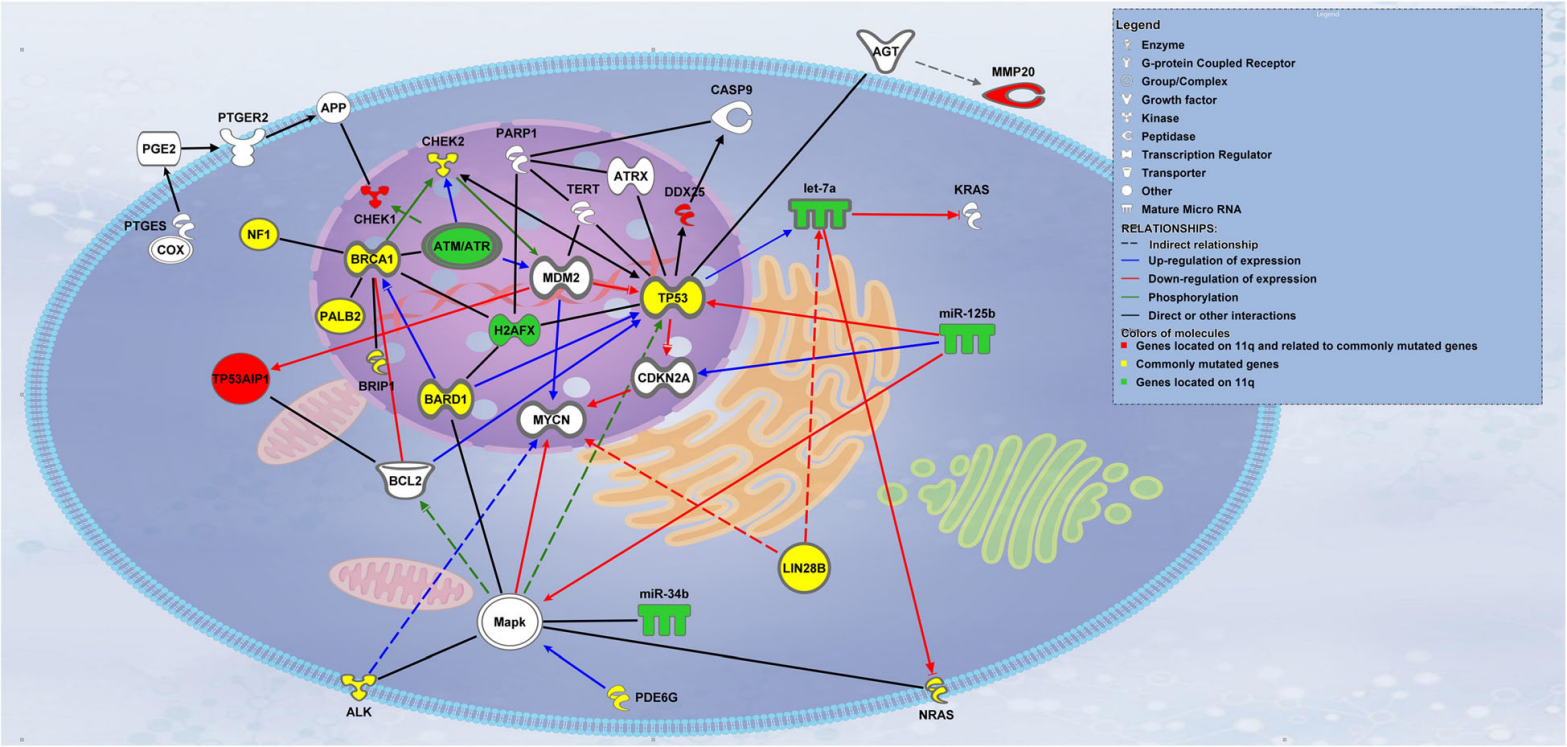
Molecular networks between genes involved in NB development by the data mining IPA software. Nine genes (*red* and *green*) on 11q recognized in regulatory networks involving *MYCN*, *MAPK*, *PGE2*, *ATM* and *p53* (experimentally described interaction only). All genes presented in this figure are highly associated with cancer (27/36 molecules, *p*-value = 2.41E-18). Where appropriate, proteins (*white*) were added by IPA software to complete the pathway. Legend contains description of basic protein functions, nature of interaction and relation to commonly mutated genes in NB or 11q deletion [10, 134]

In the particular subgroup of NBs occurring in adolescent and young adults [30], representing less than 5% of NBs and characterized by a high prevalence of SCA (35–85%) and very low incidence of MNA, the prevalence of 11q loss is high, ranging between 30 to 60% [37–39]. In this particular subgroup where *ALK* and *ATRX* alterations are also more frequent and the outcome very poor [37–40], *ATRX* mutated NBs showed a higher number of SCA including 11q deletions (Fig. 1) [37].

In 2010, Schleiermacher et al., reported genomic profiling of paired tumour samples obtained at the time of diagnosis and relapse from 17 patients with NBs. In 4 cases, 11q loss was found as an additional segmental change only present in the relapse sample. Interestingly, 2 of these 4 patients were only treated by surgery at diagnosis, suggesting that these alterations were not directly linked to the effects of the chemotherapy or irradiation. Despite many efforts, it was not possible to determine if the 11q loss observed at relapse occurred secondarily during tumour progression conferring a selective advantage to the tumour cells or was already present in a sub-clone at diagnosis [41]. More recently, a relapse-specific 11q loss was detected in 3 out of 23 NB samples by Eleveld et al., while this specific alteration was not found in 16 paired samples by Schramm et al. [42, 43].

It is now well recognized that 11q deleted NBs constitute a distinct subgroup of aggressive malignancies, but with distinct features compared to the MYC(N)-driven subset [5]. A high frequency of chromosomal breaks suggestive of a chromosomal instability [3] is one of the main features of 11q deleted NBs, reported by different groups [5, 30]. This finding suggests that one or more genes located on 11q could be involved in the chromosomal instability (CIN) phenotype, by haplo-insufficiency or by inactivation of the second allele by mutation or epigenetic modification. To address this point studies of clonal evolution on NB with 11q deletion are needed.

### 11q chromosomal deletion mapping studies

**Fig. 2:**
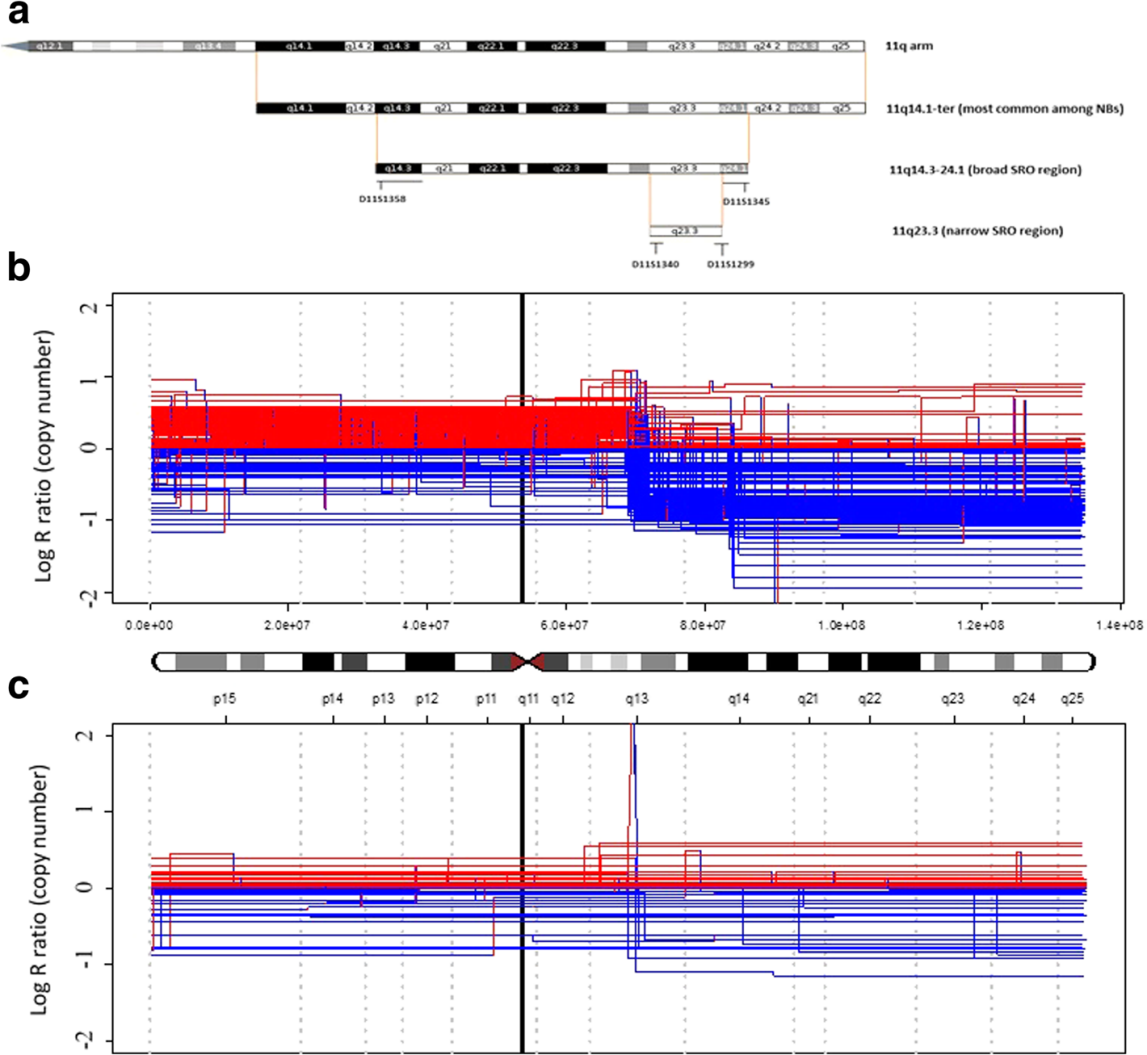
Location of deletions on 11q arm in NB tumors. **a** Location of deletions on 11q arm in NB tumors. **b** Copy number segmentation plot of chromosome 11 of 214 non-MNA high-risk tumors; horizontal lines represent single tumor dosages represented as the base 2 logarithm (y axis) of the copy number along the chromosome; vertical lines indicate breakpoints associated with gain (*red*) and loss (*blue*) of genomic regions. **c** Analogously to **b**, segmentation plot of chromosome 11 in 103 MNA tumors

Since the first recognition that 11q deletion is an important event, many studies have focused on identifying the smallest region of overlap (SRO) aiming to pinpoint the location of the gene(s) responsible for the change in cell behaviour. The main conclusion from early studies by Guo et al., Maris et al. and Schleiermacher et al. (combining in total 183 NBs) was that the majority of NBs with 11q loss have large chromosomal deletions roughly ranging from 11q14.1 to the 11qter [8, 11, 15]. The shortest deletion was reported by Schleiermacher et al. that spanned from 11q23.1 to 11qter [8]. Both Maris and Guo reported the shortest region from 11q21 to 11qter. In addition, 12 NB patients were found with intact 11qter having interstitial 11q deletion. Out of these 12 samples, 7 retained only a minor part of 11qter close to microsatellite marker D11S968 and for the remaining 5 cases, the SRO was proposed between D11S1340 (11q23.3) and D11S1299 (11q23.3) [15]. Later, after acknowledging the small number of samples used for determining the SRO, a broader SRO between D11S1358 (11q14.3) and D11S1345 (11q24.1) was proposed (Fig. 2a) [11]. More recently, segmentation data from SNP arrays obtained from the TARGET data matrix (https://target-data.nci.nih.gov/Public/NBL/copy_number_array/L3/) highlight the differences between non-MNA and MNA SCA profiles (Fig. 2b and c respectively) and spotlight a breakpoint rich region at 11q13.

Further evidence for localizing the SRO came from precise identification of constitutional 11q deletions in NB patients. A few cases of germline hemizygous partial deletion of 11q associated with NB were reported in the literature (11q23 [44]; 11q14-q22 [45]; 11q14-23 [46, 47] and 11q14.1-23.3 [48]) and seem to support the broader SRO. Interestingly, in one of these cases, the NB was multifocal [45] and in another case the NB was associated with a ganglioneuroma (GN) [47]. Using the European Cytogeneticist Association Register of Unbalanced Chromosome Aberrations database (ECARUCA database http://umcecaruca01.extern.umcn.nl:8080/ecaruca/ecaruca.jsp, date of analysis: 15.7.2016), we reviewed 132 cases with any kind of deletion between 11q14 and 11qter but no cases of NB were found. The most common abnormalities in these children were mental retardation and cranio-facial abnormalities. 11q deletions ranging from 11q24 or 11q25 to 11qter resulted in Jacobsen syndrome (OMIM 147791) but these patients did not develop NB. These data point to the conclusion that 11q deletions apparently do not have a high penetrance for NB and are not sufficient to cause it, but must be apparently accompanied by other genetic events. Additional support of such a conclusion came from early chromosomal transfer experiments carried out by Bader et al. showing that genes on 11q25 are only responsible for NB differentiation but not of proliferation capability of tumour cell lines [49].

### Functional studies and sequencing of genes at 11q

Based on the knowledge described above, different groups have used functional testing of candidate genes to try to identify the gene(s) responsible for driving the tumourigenesis. Several candidates such as *CADM1* (11q23.3, also known as *TSLC1* or *IGSF4*) [50–52], *ATM* (11q22.3) [53] and *H2AFX* (11q23.3) [5] were probed by different groups (Fig. 1). Analysis of these genes for additional alteration(s), to satisfy Knudson’s two hit hypothesis, showed no second mutation or hyper-methylation for *CADM1* [50–52], *ATM* [53] and *H2AFX* (personal unpublished data). Although clear functionality (*CADM1* and *ATM*) and associations (*CADM1, ATM, H2AFX*) of these genes were established, their relevance to the development of NB in vivo remains under question given the lack of mechanism of their complete inactivation. Recently, our lab explored the possibility that 11q could be a substitute for direct amplification of *MYCN* by upregulating its expression. Results obtained on *ATM* knock down NB cell lines showed that *MYCN* upregulation was inconsistently found, and when detected, it was not as strong as in the case of *MYCN* amplification, nor was the molecular profile similar [53]. Another reason against such hypothesis is that MNA NBs and 11q-deleted NBs appear to be 2 different molecular entities as demonstrated by mRNA expression profiling [22, 23, 54] and they appear to be mutually exclusive [11]. The reason for the latter is not yet fully understood, but it is interesting that a higher frequency of chromosomal breaks are observed in such tumours [36] perhaps pointing to the possibility of unsustainable chromosomal instability.

An additional interesting candidate frequently located near the 11q deletion breakpoints (11q13.4) is *PHOX2A* [55], a transcription factor involved in the maintenance of noradrenergic neuronal differentiation in the locus coeruleus during embryogenesis [56]. This gene is under transcriptional regulation by its homolog *PHOX2B* (4p13) [57, 58], one of the gene involved in the majority of the familial NB cases and known to be a master regulator of neural crest development [59–61]. After extensive search, no mutations in *PHOX2A* were found but authors reported lower expression of this gene in unfavourable NB [55]. Unfortunately, *PHOX2A* was not screened for hyper-methylation or small deletions for exploring other possibilities of gene inactivation.

*SDHD* (11q23.1) is a well-known tumour-suppressor gene frequently mutated in familial paraganglioma and pheochromocytoma. Both tumours derive from the sympathetic nervous system, providing dePreter et al. with a good reason for exploring the possibility that *SDHD* is the target of 11q loss. Upon detailed analysis of 31 NB cell lines and 67 NB tumours, two point mutations (frame shift and missense) were found in NB cell lines but no hyper-methylation or homozygous deletions were detected. *SDHD* mRNA expression was significantly reduced in NBs with 11q loss but functional analysis did not point to its influence on NB phenotype, prompting the group to conclude that *SDHD* is probably of lesser significance to NB development [62].

Santo et al. hypothesized that microdeletions, fusions and rearrangements of 11q23 could be involved in dysregulation of important genes. By combining SNP arrays with comparative genomic hybridization and gene expression data, they were able to identify overexpression of *FOXR1* (11q23.3) due to micro rearrangements in 3 out of 362 NB cases. Additional functional studies showed that over-expression of this gene could functionally replace *MYCN* and drive proliferation of JoMa1 cells, while repression of *FOXR1* by RNAi in HOS cells strongly inhibited proliferation and triggered apoptosis [63].

Recently, two projects using DNA exome sequencing were performed aimed at pinpointing the responsible genes in 11q deletion for the more aggressive tumour behaviour. These groups [64, 65], provided similar findings to that published by Pugh et al. where it is shown that there is a low mutation rate in the genes located at 11q and across the genome [66]. No obvious gene with second hit mutations along with 11q deletion was identified [66]. This result is in agreement with the global view of the NB mutation profile, which exhibits surprisingly only a low number of point mutations, suggesting that tumorigenesis is more reliant on larger chromosomal rearrangements. In addition there is a lack of functional studies for many of these 11q genes making proper conclusions based on gene function harder.

Although 11q has been investigated with the intent on finding tumour suppressor genes, it also harbours several oncogenes that appear to play a role in the development of NB and could present themselves as interesting targets for therapy. *Cyclin D1 (CCND1)* (11q13.3) maps into the retain side of 11q breakpoint region. Rearrangements and copy number gains resulting in over-expression have been described in both tumours and cell lines [67, 68]. CCND1 in complex with CDK4/CDK6 regulates G1/S transition and its oncogenic role in cancer has been largely associated with the inactivation of pRB. In NB, inhibition of CCND1 and its targets CDK4/CDK6 resulted in reduced cell proliferation, cell cycle arrest and neuronal differentiation [69]. Targeting CCND1 in NB through CDK4/CDK6 inhibition has attracted attention and is currently in preclinical studies [70, 71].

*NCAM* (11q23.2) is a well-known tumour marker expressed on NB whose higher expression is associated with increased metastasis at diagnosis and advanced disease [72]. Further studies in vitro and in animal models suggested that poly-sialinization of NCAM promotes dissemination of tumour cell lines by reducing the adhesiveness of cells [73]. Two enzymes, ST8SialII and ST8SialIV, carry out poly-sialinization of NCAM. Interestingly, expression of ST8SialIV but not ST8SialII is induced by retinoic acid and leads to increase in NCAM poly-sialinization [74]. In line with these observations, Al-Saraireh et al. reported that inhibition of ST8Siall by cytidine monophosphate decreases surface poly-sialinization and migration of cells in vitro [75] suggesting that NCAM or poly-sialinization enzymes could be alternative specific targets for NB treatment. Mouse model of 7 NB xenografts treated with the IMGN901 showed an objective response in 3 cell lines all having high and homogenous IHC staining for NCAM (CD56) [76] providing further evidence that NCAM could be interesting target for NB treatment.

An interesting candidate investigated was *CHK1* (11q24.2) for which pharmacological inhibitors exist and could present a novel avenue for treatment of NB [77]. Simultaneously inhibiting both Wee1 and Chk1 resulted in a marked effect on tumour size [78]. Further investigation of *CHK1* showed that its inhibition induces PP2A tumour suppressor activity resulting in de-phosphorylation of MYC and impaired cancer survival [79]. Additional studies investigating cell cycle demonstrated S phase arrest and progression towards apoptosis after *CHK1* inhibition [80]. Although CHK1 seems to play oncogenic role in NB, it also acts as a tumour suppressor as demonstrated in other cancers. CHK1 is well known to be involved in cell cycle control and chromosomal stability [81, 82]. In NB, 11q deletion could lead to haplo-insufficiency of this function proving beneficial for development of more aggressive phenotype but complete inhibition of CHK1 might prove too detrimental as it could cause mitotic catastrophe and ultimate cell death [83, 84].

### Methylation pattern of 11q

Dedicated studies exploring methylation of 11q chromosome are sparse. The most current and probably most thorough dataset is that produced by Decock et al. [85]. Although the dataset does not specifically study samples with 11q deletion, researchers excluded NBs with MNA. In addition stage 4 NBs were separated in two groups: stage 4S (NBs known to have good prognosis despite metastatic disease and low frequency of 11q deletions (4%)) and stage 4 (high risk NBs) which would further enrich the cohort for NBs with 11q deletion. Stage 4 NBs were compared to stage 4S or stage 1/2 disease [85].

Only 3 different 11q hyper-methylation sites were found when comparing HR stage 4 to stage 1 and 2 NBs and only 2 were in the vicinity of genes. One is located within 11q23 close to miR4492 whose function is unknown. The other near *USP35* (11q14) which could be another candidate gene but the significance of this finding is questionable as its location is not frequently affected by 11q deletion.

Comparing HR stage 4 to stage 4S yielded 11 hyper-methylated genes. Perhaps the most interesting of these targets is *HepaCAM* (11q24.2), a gene already known to play a tumour suppressor role in different cancers [86, 87] and already found hyper-methylated in these neoplasias [86]. Even though methylation identified only a few potential genes undergoing second hit, it is of particular interest that changes in methylation appears to affect 11q chromosome surprisingly little. These results are supported by the above-mentioned methylation and functional studies of particular genes [52, 88], where researchers were not able to find indices of hyper-methylation. Fisher et al. also obtained similar result where no major difference in expression of dysregulated genes between favourable or unfavourable NBs with 11q deletions was observed [22].

### mRNA expression profiling by DNA microarrays

The advent of DNA microarrays brought about a new approach to finding potential candidates genes associated with 11q deletion and NB aggressiveness. However, only a few dedicated mRNA expression profiling studies on NB with 11q deletion have been performed. Early results uncovered specific expression patterns associated with 11q deletion that were different to gene expression pattern of MNA or low risk NBs [54], supporting the view that NBs with 11q deletion represent a separate molecular entity. Wang et al. similarly showed that NBs with 11q deletion have a specific mRNA expression profile, distinct from NBs with normal 11q. Different genes located in the 11q13-11qter region were proposed as candidate genes, possibly implicated in the aggressive phenotype of this subgroup of NBs, but no functional analysis was performed [23]. A more detailed analysis published by Fischer et al. showed that within NBs with 11q deletion, two distinct groups with different prognosis could be established [22]. Interestingly, while favourable tumours with and without 11q deletion showed similar mRNA profiles, major downregulation of the genes located in 11q was detected only in unfavourable NBs with 11q deletion. In order to identify possible differences in the size of 11q deletion between the 11q favourable NB and 11q unfavourable groups, CGH analysis was performed showing no major difference between both groups. Altogether, these results indicate that NB with 11q loss comprises two biological and clinical distinct subgroups, they also indicate that in the unfavourable group, 11q deletion affects the expression levels of many genes located in 11q. Unfortunately, no functional studies were performed in this study thus potentially responsible gene were not identified [22].

### miRNAs of 11q

Four genes, encoding for miR4301, miR-125b-1, let-7a and miR-100 are located in the 11q23.3 SRO region (Fig. 2). *MiR-125b-1*, *let-7a* and *miR-100* are clustered together and the whole cluster is evolutionary conserved [89, 90]. In addition, *miR-100* and *miR-125b-1* show considerable sequence homology [89, 90]. MiR-125 is known to be implicated in the differentiation of neurons [91] and astrocytes [92] as well as in the plasticity of synapses [93]. In addition, Laneve et al. showed that miR-125 is also implicated in regulating NB cell growth through repression of truncated isoform of the neurotrophin receptor tropomyosin-related kinase C [94].

The let-7 miRNA family has been shown to repress clonogenic growth of NB cell lines by targeting *MYCN* mRNA, suggesting that loss of chromosome 11q could increase levels of *MYCN* [95]. Further investigation by Molenaar et al. confirmed these results and expanded the experiments to an animal model showing that lin28B is able to repress let-7 miRNA and increase MYCN protein levels inducing the development of NB [96]. This result is in accordance with observations obtained in other cancers showing that let-7a has tumour suppressor capabilities [97–100]. Closer investigation of let-7 miRNA family on chick sympathetic ganglia confirmed lin28B and let-7a functional roles described above but in contrast lin28B did not affect let-7a expression [101]. A recent study by Powers et al. again confirmed the above described observations but they further demonstrated that *MYCN* gene is able to sponge the expression of let-7 family members thereby relieving selective pressure to lose let-7 miRNA through 11q and 3p chromosomal deletions, giving possible explanation of why *MYCN* and 11q-deletions are rarely found together [102]. Association studies found correlation between SNP rs17065417 in *lin28B* with susceptibility to NB and expression of let-7 [103, 104]. Important targets include oncogene RAN and convergence of lin28B/RAN signalling on Aurora kinase A activity [104] and HACE1 [103]. In addition to *MYCN*, let-7a also binds and modulates expression of *K-RAS*, another important oncogene frequently mutated in NBs [105]. The importance of losing let-7a as a result of 11q deletion may be related to the modulation of *K-RAS* however this hypothesis remains questionable as no association of 11q deletion and expression of *K-RAS* has been found (R2, ID: GSE3960, R2 internal identifier: ps_avgpres_xtnbmaris101_u95a).

Although there is no publication describing the role of miR-100 in NBs, its association with tumour development is well established but controversial. In breast cancer, silencing of miR-100 initiated apoptosis [106] and the same effect was also observed in gastric tumour [107]. High miR-100 expression in renal carcinoma cells was associated with poorer prognosis [108], but not in hepatocellular carcinoma where downregulation of miR-100 was associated with poorer cell differentiation and shorter recurrence-free survival, while activation of miR-100 repressed metastasis [109]. In oesophageal squamous cell carcinoma [110], colorectal cancer [111], bladder carcinoma [112] and ovarian cancer [113] miR-100 downregulation was generally found to be associated with a poorer prognosis. In addition, miR-100 was shown to downregulate *ATM* and by this way, sensitize glioma cell lines to irradiation [114].

Other miRNAs located on 11q that have been studied in association with tumour biology include miR708 (11q14.1), miR34b (11q23.1) and miR34c (11q23.1). Similar to miR-100, evidence on miR708 is conflicting, with some studies reporting tumour suppressor properties in glioblastoma [115], renal cancer [116], ovarian cancer [117] and hepatocellular carcinoma [118] and others reporting oncogenic properties in bladder [119] and non-small cell lung cancers [120]. One of the most downregulated miRNAs in NB cell lines compared to normal adrenal glands was miR34b/c. Similar results were also found in a mouse NB progression model. Furthermore, treatment with 5′-AZA demethylation agent successfully re-established expression of miR34b/c, while miRNA mimic successfully decreased NB proliferation rate [121]. In support of these results in NB, it was shown that epigenetic inactivation in multiple myeloma and restoration of their expressions led to reduced cell proliferation and enhanced apoptosis [122]. Interestingly, both miRNAs appear to be under transcriptional control of p53 as they are upregulated when p53 is activated by Nutlin3a, inducing senescence [123]. Polymorphisms in the promoter region of miR34b/c are also implicated in higher risk for development of cancer [124].

Other validated or provisonal miRNAs genes located on 11q include: *miR5579*, *miR3166*, *miR1261*, *miR4300*, *miR4490*, *miR1304*, *miR1260B*, *miR3920*, *miR4693*, *miR4491*, *miR4301*, *miR4492*, *miR4493*, *miR3167* but their functions are unknown.

Investigations on expression of global miRNA profile in NB with 11q loss showed a similar picture with that of mRNA profile. Buckley et al. showed that NBs patients with 11q-deleted could be divided into high and low risk groups based on their expression profile of only 15 miRNAs. Interestingly, the miRNA profile of higher risk patients also correlated with more chromosomal abnormalities, but no other chromosomal abnormality was significantly associated with it [14].

### Haplo-insufficiency

Due to the lack of concrete findings of the above-described efforts for identifying gene(s) responsible for the increased NB aggressiveness, it was proposed that 11q deletion may be a case of haplo-insufficiency. In order to understand which pathways might be affected by haplo-insufficiency when 11q is lost, we have extracted a list of genes located on 11q14 – 11qter and a list of genes located in the SRO broad region (Fig. 2). Then using gene enrichment analysis (IPA software), we were able to identify molecular pathways most extensively affected by 11q deletion (Table 1). Certain genes identified in these pathways include: matrix metalloproteinases (known to be an important re-modellators of extracellular proteins), genes involved in bladder cancer signalling and HIF1a signalling, genes involved in immune response such as granulocyte and agranulocyte adhesion and diapedesis, leukocyte extravasation signalling and inflamasome pathway were also present. Interestingly 11q seems to also hold a high number of genes involved in apoptosis and cell death of kidney cell lines, lipid metabolism and uptake of potassium ions.

**Table 1:**
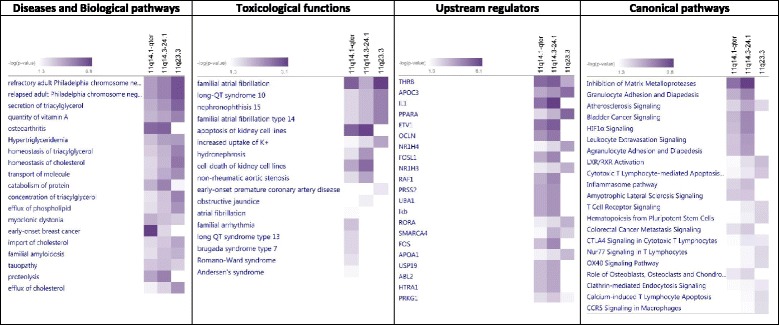
Comparison of differences in enrichment analysis between different 11q deletion segments

For the bioinformatics analysis IPA software was used. Annotated genes located on the most frequent 11q deleted region (11q14.1-qter from *PAK* to *SNX*), broad SRO (11q14.3-24.1 from *miR-4490* to *miR-100*) and narrow SRO region (11q23.3 from *CADM1* to *TRIM29*) was used. All three gene sets were ranked for Disease and Biological pathways, Toxicological Functions, Upstream regulators and Canonical pathways (in table only significant are shown). Fisher exact test was used for determination of enrichment and *p*-value less than 0.05 was used as significant. The rank score represents the ratio of 11q deleted genes contained in a specific network in comparison to the whole list of genes involved in the same specific network

### The timing of the 11q and its location in the different cellular components of neuroblastic tumours

Precise timing of 11q deletion is not yet fully understood. It’s increasing frequency with stage, being 8% in stage 1 and 52% in stage 4 [27] and older age of NB onset [5], could suggest a late stage event. Other hypothesis of being early stage event persisting after birth is possible as well. Interestingly, a recent research analysing of stage 4 and 4S NB, suggests that 11q loss might appear at early stages of metastatic tumorigenesis, preceding 3p loss [125]. CGH analysis of ganglioneuroblastoma (GNB) showed no 11q deletion, suggesting that this event might occur rarely in this less aggressive NB subgroup [126]. Similar results were obtained by Coco et al. and Bourdeaut et al. where CGH did not show any abnormalities at chromosome 11 in ganglioneuroma (GN) and GNB intermixed, while 11q loss was detected in NB [127, 128].

Although neuroblastic tumours are very heterogeneous tumours composed of variable proportion of neuroblastic and Schwannian stromal cells, the common origin of both component and the neoplastic nature of Schwann cells were highly debated [127–132]. In 2008, Bourdeaut et al. were able to show by using X-linked inactivation and CGH, that neuroblastic and Schwannian stromal cells develop from the same GNB progenitor but later undergo separate development [128]. In this study, while all neuroblastic components showed chromosomal imbalance (4/6 with 11q loss), no alteration was observed in the Schwann cells area, pointing to the likely possibility that 11q is acquired at the stage where the neuroblastic component is already differentiated from Schwann cells stroma [128]. More recently, Angelini et al. reported that ganglion cells, but not Schwann cells, of nodular GNB showed similar genetic alterations detected in the neuroblastic component. In this study, 5 of 8 nodular GNB showed 11q loss in neuroblastic cells. When tested, 11q loss was also detected in the ganglioneuromatous component of the tumour, but not in Schwann cells. Altogether, the results obtained from using SNP arrays and FISH suggest that Schwann cells have a different origin and are not clonally related to the other compounds [133].

## Conclusions

During the past 25 years, substantial efforts have been invested in trying to understand the molecular changes that lead to more aggressive NB tumours. As very few mutations have been detected despite large international efforts, recurrent patterns of NCA or SCA suggest that NB is most probably a cancer driven by copy number rather than by specific mutations. Using classical cytological methods, 11q deletion was identified as one of the main events associated with poorer prognosis. Further molecular characterization using LOH and microarrays CGH or SNPs, pinpointed a possible SRO within 11q. The observation that 11q is never lost on both chromosomes lead to the suggestions that vitally important genes must be present on the remaining 11q, but that the second hit needed would logically be caused by a smaller localized mutation or methylation event. However, research aimed at pinpointing the culprit gene through sequencing and methylation has yielded unsubstantial results.

Due to the unsuccessful efforts despite using the latest sequencing technologies, it seems unlikely that classic single unifying tumour suppressor gene explanation exists for 11q deletion. Because of that, it has been proposed that 11q deletion might be a case of haplo-insufficiency. An alternative approach for identifying the 11q locus(i) responsible for the 11q loss phenotype might be possible with CRISPR/Cas9 genomic editing. Although only a few publications exist for this application, it has demonstrated that creating gross deletions using a two guided RNA system is possible. A deletion of up to 1 Mbp has an efficiency of around 1 to 2%, sufficient for the generation of such clones with proper automatization equipment. It was demonstrated that deletions of up to 60 Mbp are possible. Such technology could prove a powerful tool for studying haplo-insufficiency by deleting progressively narrower regions and determining their contribution to tumorigenesis or aggressiveness at every stage by standard cytological methods. Developing such models of 11q deletions would be an essential first step to defining therapeutic vulnerabilities for this group of patients.

## Abbreviations

CGH: Comparative genomics hybridization
CIN: Chromosomal instability
DNA: Des oxyribonucleic acid
ECARUCA: European Cytogeneticist Association Register of Unbalanced Chromosome Aberrations
EFS: Event free survival
FISH: Fluorescence in situ hybridization
GN: Ganglioneuroma
GNB: Ganglioneuroblastoma
HR: High risk
IPA: Ingenuity Pathway Analysis
LOH: Loss of heterozygosity
MNA: MYCN amplification
NB: Neuroblastoma
NCA: Numerical chromosome aberrations
OS: Overall survival
SCA: Segmental chromosomal aberrations
SNP: Single nucleotide polymorphism
SRO: Smallest region of overlap
